# Long COVID Syndrome, Mortality and Morbidity in Patients Hospitalized with COVID-19 From 16 Countries: The World Heart Federation Global COVID-19 Study

**DOI:** 10.5334/gh.1452

**Published:** 2025-08-01

**Authors:** Karen Sliwa, Kavita Singh, Kalyani Nikhare, Dimple Kondal, Lana Raspail, Meetushi Jain, Shahin Akter, Shamim Hayder Talukder, Toru Kato, Silvia Bertagnolio, Jamie Rylance, Amitava Banerjee, Jagat Narula, Daniel Pineiro, Pablo Perel, Dorairaj Prabhakaran, The WHF COVID-19 Long Study Collaboration

**Affiliations:** 1 Cape Heart Institute, Department of Medicine and Cardiology, Groote Schuur Hospital, Faculty of Health Sciences, University of Cape Town, South Africa; 2 World Heart Federation, Geneva, Switzerland; 3 Public Health Foundation of India, New Delhi, India; 4 Centre for Chronic Disease Control, New Delhi, India; 5 Heidelberg Institute of Global Health, Heidelberg University, Germany; 6 Eminence, Bangladesh; 7 NHO Tochigi Medical Center, Japan; 8 World Health Organization, Geneva, Switzerland; 9 University College London, United Kingdom; 10 University of Texas Health Science Center, Houston, United States of America; 11 University of Buenos Aires, Argentina; 12 Department of Non-communicable Disease Epidemiology, London School of Hygiene & Tropical Medicine, United Kingdom; 13 Centre for Chronic Disease Control, New Delhi, India and Public Health Foundation of India, New Delhi, India; 14 London School of Hygiene & Tropical Medicine, United Kingdom

**Keywords:** COVID-19, registry, cohort, survey, coronavirus, cardiovascular disease

## Abstract

**Background:**

Long-term adverse consequences of the COVID-19 infection affect many organ systems, which requires comprehensive understanding of the disease burden and determinants of persistent long COVID-19 symptoms in diverse population. However, data on long COVID complications are sparse, particularly from low- and middle-income countries (LMICs). The World Heart Federation (WHF) global study assessed the incidence of vascular complications, persistent long COVID symptoms and factors associated with mortality and major adverse cardiovascular events (MACE) among patients with COVID-19 up to one year after hospitalization.

**Methods:**

We recruited a total of 2535 patients hospitalized with COVID-19 and followed up to one-year post-hospital discharge. We collected data on long COVID symptoms, quality of life, and clinical outcomes, including new onset diseases, MACE, and mortality at 1-, 3-, 6-, and 9–12 months post-discharge. Descriptive and generalized estimating equation (GEE) regression analysis was performed to assess the factors associated with mortality and MACE.

**Findings:**

The majority of participants were recruited from LMICs (64%) and male (56%) with a mean (SD) age of 59.5 (20.0) years. Among those tested for COVID-19 strain (52%), Omicron strain was the most prevalent (98%). The follow-up rate at one year was 90%. Over half of the participants (56%) reported experiencing at least one major long COVID symptom (fatigue, breathlessness, anxiety, chest pain, and palpitations) at 1-month, and one-quarter participants reported persistent long COVID symptoms at 9–12 months. On the EQ-5D scale, 49% reported difficulties in usual activities, 33% reported anxiety/depression, and 23% reported problems in mobility within the first 6 months. The most frequent new-onset illnesses were pulmonary embolism (8%), kidney disease (4%), and hypertension (3%). The cumulative all-cause mortality rate was 15% (n = 382) at one-year post-discharge. Long COVID symptoms were more common among females, individuals with pre-existing comorbidities, and those with more severe acute illness. Age, obesity, ICU admission, and underlying cardiovascular or pulmonary disease were associated with increased risk of mortality and MACE.

**Conclusion:**

The study showed a substantial burden of mortality and morbidity, and a quarter of patients reported at least one persistent long COVID symptom after one year. Our findings underscore the need for early identification and management of long COVID symptoms in LMICs.

## Introduction

Long COVID is a multi-systemic disease, affecting people across the lifespan. A few studies with 2–3 years of follow-up have shown a persistent risk of developing long-term clinical sequelae after COVID-19 infection (1,2). Evidence so far suggests that the severity of acute COVID-19 illness is the most common risk predictor for long COVID (3). Effects of COVID-19 hospitalization on a range of self-care and health-related behaviours are therefore expected, including long-term symptoms such as breathlessness, multi-morbidity, fatigue, and physical inactivity. The acute phases of COVID-19 infection, in particular the delta- and beta-strains are well studied; however, there is limited evidence on the acute and long-term (>3–6 months) effects of the Omicron strain-COVID infection (i.e. the burden of long-term health loss) (1). Previous studies reported huge societal and financial burden associated with the Long COVID symptoms that necessitate an urgent public health need to understand the underlying mechanisms driving the sequelae of the COVID-19 infection (long COVID) especially in the post-hospitalization survivors to improve treatment and prevention of long COVID for future waves of pandemic (4,5). While some COVID-19 survivors will have a full recovery, others may have or may develop other chronic conditions, mental health issues, and often a combination of multi-morbidities. The challenges are thus to understand the magnitude of the burden of this post-COVID-19 infection (long COVID) and hospitalization in specific at-risk groups; the underlying mechanisms and biomarkers to predict future risk, the most efficacious and cost-effective pharmacological and non-pharmacological interventions, and to construct long-term integrated care pathways required to tackle future waves of COVID-19 and other pandemics. Importantly, although the trajectories are likely to be heterogeneous across high-, middle-, and low-income countries, most of the studies have been conducted in high-income countries or focused in a single country or region (1,3).

The World Heart Federation (WHF) established a cohort of more than 5000 hospitalized COVID-19 patients from 23 countries around the world during the early phases of the COVID-19 pandemic in June 2020. The study aimed to describe the short-term cardiovascular manifestations and cardiovascular risk factors in patients with COVID-19 (6,7). We reported important findings with a relatively young population and a 30-day mortality of 15.1%, of which 20% were due to sudden cardiac death (7).

The purpose of the WHF COVID-19 Long-Term Follow-Up Study (conducted from January 2022 to August 2023) was to describe the COVID-19 pandemic in the new epidemic context (when new strains were prevalent and a larger proportion of the population were vaccinated), with a special focus on the long-term (up to 9–12 months) outcomes since hospital discharge.

The overall aim of the WHF COVID-19 long-term follow-up study was to describe long-term outcomes for survivors who had been hospitalized for COVID-19 infection. Our objectives were as follows:

1. To determine the short- (1 and 3 month), medium- (6 month), and long-term (9–12 month) sequelae to COVID-19, including re-hospitalization, mortality, persistent symptoms, impact on physical function, psycho-social consequences, quality of life, and cost of care.
2. To assess associations between short- to long-term outcomes and demographics, ethnicity, socioeconomic status, lifestyle factors, pre-existing comorbidities, and severity of acute COVID-19 illness.

## Methods

### Study design and setting

This is a global, multi-centre prospective cohort study involving COVID-19 hospitalized patients across low-, middle-, and high-income countries. Twenty-six hospitals (in 16 countries) from the WHF network agreed to participate, recruit COVID-19 hospitalized patients prospectively, and follow them up for one-year post-hospital discharge. The patient recruitment began in January 2022, with subsequent follow-ups scheduled after their discharge from the hospital. We conducted follow-up assessments at 1-, 3-, 6, and 9–12 months post-hospital discharge and completed telephonic follow-up assessments of all eligible patients in January 2024. The study was conducted in partnership with the World Health Organization (WHO), which offered scientific expertise support throughout the study duration for data collection and analysis. Ethics approval for this study was obtained from the University of Cape Town, South Africa, and the Public Health Foundation of India, India (Coordinating Centres). In addition, each participating site also obtained local institutional ethics approval to recruit patients and conduct follow-up for up to one year after discharge. National regulatory clearances were also obtained as required. Eligible patients were thoroughly informed about the study purpose, the one-year telephonic follow-up process, associated risks, and potential benefits of participating in the study. The patients who agreed to participate provided written informed consent.

### Study population

The enrolment criteria were all adult patients (≥18 years) who were hospitalized with a confirmed COVID-19 diagnosis and discharged from the hospital facility were eligible for inclusion, regardless of the length of hospital stay. There was no minimum stay requirement; both short-stay and long-stay hospitalizations, including those requiring intensive care, were included. Patients who were unable or refused to provide consent were excluded.

#### Data collection and measurements

Research data were collected using study CRFs (case report forms) that have been developed in collaboration with the WHO and recorded in the REDCap database. Two CRFs were developed: the baseline CRF included data on socio-demographics (age, sex, education, ethnicity, smoking, and pregnancy status), pre-existing cardiovascular and non-cardiovascular comorbidities, pre-admission medications, vital signs upon hospital admission (including symptom onset, blood pressure, temperature, oxygen, respiratory rate, shortness of breath, and APVU responsiveness scale), COVID-19 diagnosis, symptoms, treatment, and supportive care administered, laboratory investigations (blood results, ECG, Echocardiography, and Chest X-ray if performed), SARS-CoV-2 sequencing tests performed during hospitalization, COVID-19 vaccination status, and outcomes at discharge (mortality and major adverse cardiovascular events (MACE)). The baseline data were collected using patients’ medical records, clinical notes, and discharge summary using study CRF.

The follow-up CRF included data on clinical outcomes, persistent long COVID symptoms, new onset of diseases, reinfection, re-hospitalization, COVID vaccination status, quality of life using the EuroQol 5 Dimension (EQ-5D) scale, working status, and cost of care. Although there is no agreed upon standard case definition of long COVID, we defined long COVID as persistence of COVID-19 symptoms (fatigue, loss of smell/taste, cough, joint pain, shortness of breath, chest pain) over an extended period that develops during or following a confirmed case of COVID-19 and continues for more than 28 days. This is similar to the definition of the Centers for Disease Control and Prevention’s (CDC) ‘Post-COVID conditions’ (8). The follow-up CRF was administered telephonically four times at 1-, 3-, 6-, and 9–12-months after hospital discharge. The follow-up visits were calculated based on the date of hospital discharge. Data were collected within seven days of the scheduled follow-ups at 1-, 3-, 6- and 9–12 months post hospital discharge. A health worker (junior medical doctor or nurse) was trained at each participating site to collect data via telephone using the CRFs. The PHFI staff provided online training for data collection and follow-up procedures. To ensure maximum follow-up, the PHFI data team reviewed the data weekly and sent reminders for patient follow-up to each site. When patients could not be reached for follow-up, the health worker contacted family members or the clinical departments where they had received routine treatment. If the patients were admitted to the same hospitals for a clinical event, hospital records were reviewed to determine the cause of admission or death. The sites contacted patients during a 7-day window period to gather data, and patients who could not be reached after numerous attempts were considered lost to follow-up.

Information on new-onset cardiovascular events and chronic conditions during follow-up was collected through structured telephone interviews using standardized case report forms. When participants were reported to have died, study staff contacted family members or caregivers to gather details on the circumstances and timing of death. Where feasible, hospital records were reviewed to validate the cause of death. However, medical death certificates were not uniformly available across all sites. In the absence of clinical documentation, verbal information from family members was used to classify the likely cause of death, although a formal verbal autopsy protocol was not applied consistently across sites.

**Figure 1:**
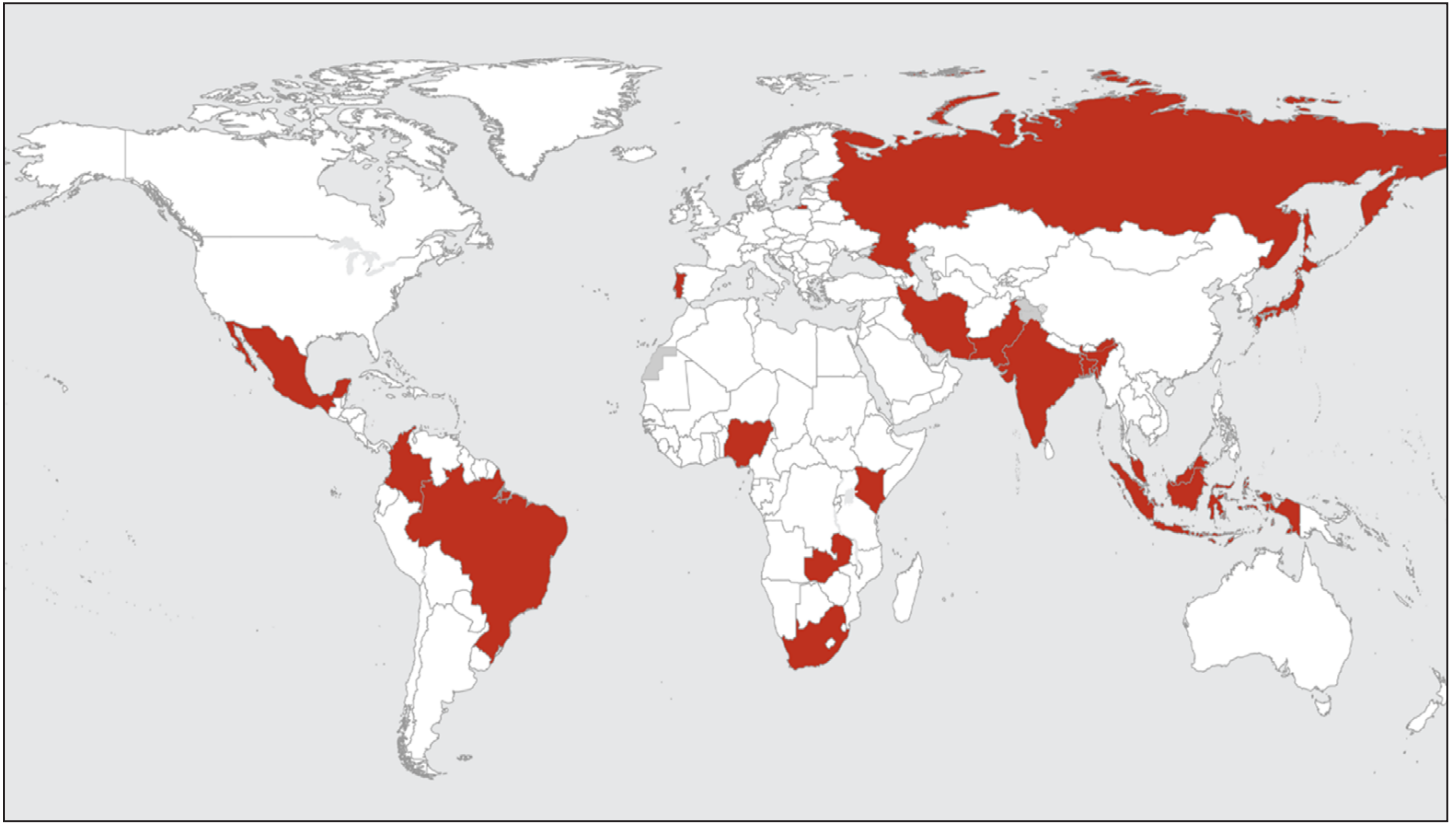
Countries participating in WHF COVID-19 Long-term Follow-up Study.

The data were entered into the online secured electronic clinical data management system (REDCap), which was overseen by the Public Health Foundation of India (PHFI). Each study site designated a study investigator and research coordinator were trained for study procedures, recruitment, consent process, data collection using study CRFs, and data entry on REDCap. The data management team at PHFI conducted monthly data reviews, and any data queries or concerns were communicated to the site coordinators. Figure 1 shows the location of the participating countries on the world map for the WHF COVID-19 Long-term follow-up study.

#### Study outcomes

The study evaluated the following clinical outcomes at discharge, 1-, 3-, 6-, and 9–12 months after discharge among survivors of COVID-19 hospitalizations: 1) persistent long COVID symptoms; 2) new onset chronic conditions; 3) CVD complications and mortality; and 4) health related quality of life as measured by the EQ-5D scale. Persistent long COVID symptoms were defined as fatigue, anxiety, memory or concentration problems, muscle weakness, dyspnea, palpitation, chest pain, loss of smell, and loss of taste following discharge. The new-onset chronic conditions included hypertension, diabetes, respiratory diseases, cardiovascular conditions (myocardial infarction, heart failure, atrial fibrillation, and stroke), and kidney diseases, which were self-reported by study participants. CVD complications included major adverse cardiovascular events (MACE), i.e., newly diagnosed heart attack, heart failure, atrial fibrillation, stroke, deep vein thrombosis, and pulmonary embolism after discharge. Causes of deaths were categorized into sudden cardiac death, other cardiovascular diseases, and non-cardiovascular diseases. Sudden cardiac death (SCD) was defined as an unexpected death that occurred within one hour of symptom onset in witnessed circumstances or within 24 hours of being last observed alive and without symptoms in unwitnessed circumstances (9). Quality of life was measured by the EQ-5D scale, which assess quality of life across five domains: mobility, self-care activities, usual activities, pain/discomfort, and anxiety/depression (10). Fatigue was assessed using a scale of 0–10. Additionally, data on healthcare utilization, employment status, and the cost associated with care utilization after discharge were collected.

#### Sample size and power calculation

The sample size was calculated assuming the expected proportion of long-term symptoms as 10% with a relative precision of 5% (2% absolute precision) at a significance level of 0.05. The required sample size was 2341, adjusting for an 80% follow-up rate. The sample size estimation was not stratified by region or ethnicity, as recruitment was determined by the feasibility and capacity of sites participating during the pandemic period.

#### Statistical analysis

The demographic characteristics and vital signs and symptoms at baseline were presented overall and by overall survival status. The data are presented as mean (SD) or median (IQR) for continuous variables and expressed as absolute values along with percentages for categorical variables. The t-test was used for continuous variables (normally distributed) and the chi-square test for categorical variables to compare the difference in demographic and clinical characteristics among COVID-19 survivors and non-survivors. Outcomes (alive, died, re-hospitalized, lost to follow-up), persistent long COVID symptoms, and new-onset chronic conditions for each of the study time points were presented as proportions. To determine the risk factors associated with mortality and MACE, we used a Generalized Estimating Equation (GEE). The GEE analysis accounts for the correlation of observations over the time points. The log-binomial models with an exchangeable correlation matrix were used. Wherever the log-binomial models failed to converge, we used Poisson models (11). For each covariate, we ran separate GEE models which included the outcome variable (mortality), the exposure variable (socio-demographic characteristics, comorbidities, etc.), time (categorical), and site (fixed effect). The covariates included were socio-demographic (age, sex, ethnicity, region, World Bank income groups, smoking, and BMI), comorbidities (hypertension, coronary artery disease, stroke, heart failure, renal disease, COPD, asthma, diabetes, tuberculosis, and HIV), oxygen therapy, and ICU admission. Similar GEE analyses were done with MACE as an outcome. Relative Risks (RR) with 95% confidence intervals were reported separately for mortality and MACE for each covariate. All significance tests were two-sided, and a p-value of less than 0.05 was considered statistically significant. All statistical analyses have been performed using STATA version 16.

### Role of funding source

The WHF COVID-19 Global Cardiovascular Disease and COVID-19 Long-term study is funded by World Heart Federation, Geneva, Switzerland, World Health Organization, Geneva, Switzerland, and Sanofi, France. The study was conducted independently without any direct involvement of the funders. The funding agencies were not involved in the study design, conduct, analysis or reporting of the results or manuscript preparation.

## Results

### Baseline characteristics of study participants at admission

Twenty-six hospitals from 16 countries (LIC = 1, LMIC = 15, UMIC = 7, HIC = 3) recruited 2535 hospitalized COVID-19 patients (LIC = <1%, LMIC = 63%, UMIC = 21%, HIC = 15%) from January 2022 to August 2023. Follow-ups for all eligible patients were completed in January 2024, with a follow-up rate of 90% (2274/2535). The socio-demographic characteristics of the study participants based on their survival status are reported in Table 1. The mean (SD) age of all recruited participants was 59.5 (20.0) years, 56% were male, and 77% were Asian. Non-survivors had a significantly higher mean (SD) age of 76.2 (13.9) years compared to survivors: 56.6 (19.4) years. The mean (SD) Body Mass Index (BMI) was 24.5 (4.2) kg/m^2^, and it was similar for both survivors (24.5 (4.1)) and non-survivors (24.2 (4.7)). Approximately a quarter of the participants were overweight (BMI 25–29), and 7.4% were categorized as obese (BMI ≥30). However, BMI information was not available for 15.8% of the participants. More than half were never smokers with 8% current smokers and 24.3% were ex-smokers. A large majority had either secondary school or university education.

**Table 1:** Participants’ socio-demographic characteristics, COVID-19 symptoms and vital signs at baseline by survival status.


	OVERALL	NON-SURVIVORS	SURVIVORS	p-VALUE

	N = 2535	N = 382 (15%)	N = 2153 (85%)	

**Sex**				0.006

Male	1408 (55.5%)	236 (61.8%)	1173 (54.5%)	

Female	1125 (44.4%)	146 (38.2%)	979 (45.5%)	

**Age, mean (SD)**	59.5 (20.0)	76.2 (13.9)	56.6 (19.4)	<0.001

**Age, category**				<0.001

<50 years	812 (32.0%)	23 (6.0%)	789 (36.6%)	

51–60 years	422 (16.6%)	20 (5.2%)	402 (18.7%)	

61–70 years	496 (19.6%)	57 (14.9%)	439 (20.4%)	

>70 years	805 (31.8%)	282 (73.8%)	523 (24.3%)	

**Ethnic Origin**				<0.001

Caucasian	17 (0.7%)	1 (0.3%)	16 (0.7%)	

Hispanic	168 (6.6%)	64 (16.8%)	104 (4.8%)	

Black	69 (2.7%)	14 (3.7%)	55 (2.6%)	

Middle Eastern	101 (4.0%)	13 (3.4%)	88 (4.1%)	

Asian	1951 (77.0%)	265 (69.4%)	1686 (78.3%)	

Other	229 (9.0%)	25 (6.5%)	204 (9.5%)	

**WHO Region (Total sites = 26)**				<0.001

Europe (n = 2)	106 (4.2%)	18 (4.7%)	88 (4.1%)	

Asia Pacific (n = 4)	576 (22.7%)	59 (15.4%)	517 (24.0%)	

Latin America (n = 4)	223 (8.8%)	67 (17.5%)	156 (7.2%)	

Middle East (n = 1)	100 (3.9%)	13 (3.4%)	87 (4.0%)	

Southeast Asia (n = 10)	1378 (54.4%)	205 (53.7%)	1173 (54.5%)	

SSA (n = 5)	152 (6.0%)	20 (5.2%)	132 (6.1%)	

**World Bank Income Groups (Total sites = 26)**				0.011

LIC (n = 1)	20 (0.8%)	4 (1.0%)	16 (0.7%)	

LMIC (n = 15)	1610 (63.5%)	232 (60.7%)	1378 (64.0%)	

UMIC (n = 7)	525 (20.7%)	101 (26.4%)	424 (19.7%)	

HIC (n = 3)	380 (15.0%)	45 (11.8%)	335 (15.6%)	

**Body Mass Index (kg/m^2^), mean (SD)**	24.5 (4.2)	24.2 (4.7)	24.5 (4.1)	0.32

**Body Mass Index (kg/m^2^) categories**				0.11

Underweight (<18)	77 (3.0%)	16 (4.2%)	61 (2.8%)	

Normal weight (18–24)	1275 (50.3%)	170 (44.5%)	1105 (51.3%)	

Overweight (25–29)	596 (23.5%)	67 (17.5%)	529 (24.6%)	

Obese (≥30)	187 (7.4%)	23 (6.0%)	164 (7.6%)	

Unknown	400 (15.8%)	106 (27.7%)	294 (13.7%)	

Confirmed case of COVID-19 infection	2528 (99.7%)	379 (99.2%)	2149 (99.8%)	0.040

Diagnosed by using RT-PCR	2212 (87.3%)	313 (81.9%)	1899 (88.2%)	0.002

Laboratory test confirmation (antigen test or molecular test- Positive result	2259 (89.1%)	332 (86.9%)	1927 (89.5%)	0.31

**Presence of signs and symptoms suggestive of COVID-19**	2360 (93.1%)	354 (92.7%)	2006 (93.2%)	0.69

A history of self-reported fever	1875 (74.0%)	269 (70.4%)	1606 (74.6%)	0.081

Cough	1955 (77.1%)	283 (74.1%)	1672 (77.7%)	0.12

Dyspnoea (shortness of breath) OR Tachypnoea	1316 (51.9%)	261 (68.3%)	1055 (49.0%)	<0.001

Clinical suspicion of ARI despite not meeting criteria above	252 (9.9%)	49 (12.8%)	203 (9.4%)	0.037

**Median time from symptom onset to admission (IQR) in days**	2 (2, 4)	3.0 (2.0, 5.0)	2.0 (2.0, 4.0)	0.009

**Heart rate (beats/min), mean (SD)**	90.2 (16.7)	91.2 (17.5)	90.1 (16.6)	0.23

**Bradycardia (HR <60 bpm), mean (SD)**	52.7 (5.5)	54.4 (4.0)	52.3 (5.8)	0.35

**Tachycardia (HR >100 bpm), mean (SD)**	114.8 (12.8)	116.2 (12.4)	114.6 (12.8)	0.29

**Oxygen, first reading (mmHg), mean (SD)**	77.5 (46.2)	78.1 (34.1)	77.2 (52.1)	0.90

**SpO2 level%, mean (SD)**	94.9 (4.9)	93.1 (6.7)	95.2 (4.4)	<0.001

**Oxygen therapy**	631 (24.9%)	181 (47.4%)	450 (20.9%)	<0.001

**Systolic BP (mmHg), mean (SD)**	125.8 (21.4)	124.6 (22.1)	126.0 (21.3)	0.24

**Diastolic BP (mmHg), mean (SD)**	76.6 (12.4)	74.7 (12.7)	76.9 (12.3)	0.001

**Respiratory rate (breaths/min), mean (SD)**	20.5 (4.1)	21.4 (4.3)	20.2 (4.1)	<0.001

**Shortness of Breath (SOB)**				<0.001

SOB <100 m	329 (13.0%)	79 (20.7%)	250 (11.6%)	

SOB 100–500 m	95 (3.7%)	29 (7.6%)	66 (3.1%)	

SOB >500 m	26 (1.0%)	4 (1.0%)	22 (1.0%)	

Unknown	955 (37.7%)	157 (41.1%)	798 (37.1%)	

**AVPU: responsiveness scale**				<0.001

Alert	1694 (66.8%)	215 (56.3%)	1479 (68.7%)	

Responsive to verbal stimulation	258 (10.2%)	44 (11.5%)	214 (9.9%)	

Responsive to painful stimulation	548 (21.6%)	114 (29.8%)	434 (20.2%)	

Unresponsive	35 (1.4%)	9 (2.4%)	26 (1.2%)	

Length of hospital stay (Mean, SD)	11.3 (25.1)	15.4 (33.7)	10.9 (23.9)	0.006

Length of hospital stay (Median, IQR)	8 (5, 11)	10 (7, 15)	7 (5, 11)	<0.001

ICU admission	309 (12.2%)	94 (24.6%)	215 (10.0%)	<0.001


#### COVID-19 symptoms, vital signs, and comorbidities

Overall, 99.7% of cases were confirmed as COVID-19 infections, with 87.3% diagnosed through RT-PCR tests, of which 89.1% reported positive test results (Table 1). A majority of the participants (51.6%) underwent sequencing tests, and the most prevailing variant of concern detected was Omicron (B.1.1.529, designated Oct 2021), accounting for 98.2% of those tested.

Most commonly reported symptoms on admission were cough (77.1%) and fever (74.0%). Additionally, over half of the participants (51.9%) experienced difficulty in breathing, either in the form of dyspnoea or tachypnoea. The presence of dyspnoea or tachypnoea was notably higher among non-survivors (68.3%) in comparison to survivors (49.0%) (p-value < 0.001). The median duration from the onset of symptoms to admission was two days (IQR: 2 to 4).

Overall, one-fourth of participants received oxygen therapy, with a higher proportion of non-survivors (47.4%) receiving oxygen therapy as compared to survivors (20.9%) (p-value < 0.001). The most commonly reported cardiovascular comorbidities were hypertension (54.4%), coronary artery disease (14.0%), heart failure (7.1%), stroke (5.3%), and atrial fibrillation (4.3%). Non-survivors reported a higher prevalence of hypertension (66.0% vs 52.4%, p-value < 0.001) and coronary artery disease (19.4% vs 13.1%, p-value < 0.001) at baseline. Regarding non-cardiovascular comorbidities, diabetes (37.9%), chronic kidney disease (12.9%), chronic pulmonary disease (9.9%), and asthma (8.8%) were commonly reported. Non-survivors reported higher prevalence for these conditions: diabetes (46.1% vs 36.4%, p-value = 0.001), chronic kidney disease (23.0% vs 11.1%, p-value < 0.001), and chronic pulmonary disease (18.3% vs 8.4%, p-value < 0.001) (Supplementary file, Table A).

#### COVID-19 vaccination status

Two-thirds of the participants (66.6%) indicated COVID-19 vaccination at the time of hospital admission, whereas 55% of participants received at least two doses of COVID-19 vaccination. During the follow-up period, more than half of the participants reported being vaccinated against COVID-19 (1-month = 48.0%; 3-month = 52.5%; 6-month = 51.6%; and 9–12-month = 52.8%) (Supplementary file, Figure A). Overall, 69.7% of participants reported receiving the COVID-19 vaccine during the follow-up period.

#### Clinical outcomes and long COVID symptoms during follow-up

Among the 2535 participants recruited, 128 (5%) participants had in-hospital mortality, while 254 (10%) additional deaths were reported during the one-year follow-up period. The overall mortality rate was 15% (382/2535). Sudden cardiac death emerged as a major cause of death (44%, n = 113), while other causes included cardiovascular diseases causes such as myocardial infarction, stroke, heart failure, pulmonary embolus (24%, n = 60), as well as non-cardiovascular diseases causes like respiratory failure (32%, n = 81). Out of the 2535 participants, 2274 (90%) completed at least one follow-up assessment, whereas 10% (n = 261) were lost to follow-up throughout one year (Table 2).

**Table 2:** Clinical outcomes, Long COVID symptoms, and new onset diseases since discharge over the follow-up period.


	1 MONTH FOLLOW-UP	3 MONTH FOLLOW-UP	6 MONTH FOLLOW-UP	9–12 MONTH FOLLOW-UP

Outcomes at follow-up visits	N = 2070	N = 1974	N = 1998	N = 1916

Alive	1808 (87.3%)	1786 (90.5%)	1725(86.3%)	1683 (87.8%)

Re-hospitalized	22 (1.1%)	12 (0.6%)	20 (1.0%)	11 (0.6%)

Death	28 (1.4%)	41 (2.1%)	98 (4.9%)	87 (4.5%)

Unknown/Loss-to-follow-up	210 (10.1%)	133 (6.7%)	151 (7.6%)	124 (6.5%)

**Causes of Deaths**				

Sudden cardiac death	8 (0.4%)	9 (0.5%)	65 (3.3%)	31 (1.6%)

Other cardiovascular cause	7 (0.3%)	12 (0.7%)	16 (0.8%)	24 (1.2%)

Non-cardiovascular cause	13 (0.6)	20 (1.0%)	16 (0.8%)	32 (1.7%)

**Persistent long COVID symptoms**				

At least one long COVID symptom	1154 (55.7%)	724 (36.7%)	908 (45.4%)	471 (24.6%)

Fatigue (worn out/lacking energy or zest)	816 (39.4%)	443 (22.4%)	423 (21.2%)	277 (14.5%)

Feeling more anxious/worrying	371 (17.9%)	213 (10.8%)	177 (8.9%)	150 (7.9%)

Breathlessness	219 (10.6%)	96 (4.9%)	78 (3.9%)	93 (4.9%)

Problems with memory, concentration or decision making	176 (8.5%)	293 (14.8%)	623 (31.2%)	165 (8.6%)

Chest pain	174 (8.4%)	85 (4.3%)	85 (4.3%)	98 (5.1%)

Palpitations	171 (8.3%)	92 (4.7%)	173 (8.7%)	163 (8.5%)

Myalgia (muscles aches)	72 (3.5%)	34 (1.7%)	42 (2.1%)	46 (2.4%)

Anosmia (no sense of smell)	14 (0.7%)	6 (0.3%)	4 (0.2%)	4 (0.2%)

No sense of taste	24 (1.2%)	7 (0.4%)	21 (1.1%)	20 (1.0%)

**Intensity of fatigue**				

Mean (SD)	4.0 (2.3)	3.4 (2.1)	3.3 (2.1)	3.1 (2.1)

None	295 (14.3%)	338 (17.1%)	319 (16.0%)	331 (17.3%)

Mild Fatigue (less than 5)	585 (28.3%)	752 (38.1%)	853 (42.7%)	892 (46.6%)

Moderate Fatigue (5)	416 (20.1%)	503 (25.5%)	326 (16.3%)	232 (12.1%)

Severe Fatigue (more than 5)	519 (25.1%)	185 (9.4%)	209 (10.5%)	205 (10.7%)

**New onset of disease since discharge**				

Onset of any 1 disease	223 (10.8%)	90 (4.6%)	65 (3.3%)	43 (2.2%)

Pulmonary embolism (PE, ‘Clot in lung’)	127 (6.1%)	35 (1.8%)	7 (0.4%)	4 (0.2%)

Kidney problems	45 (2.2%)	21 (1.1%)	18 (0.9%)	7 (0.4%)

New onset hypertension	22 (1.1%)	15 (0.8%)	19 (1.0%)	15 (0.9%)

New onset diabetes	17 (0.8%)	7 (0.4%)	6 (0.3%)	3 (0.2%)

Heart Failure	10 (0.5%)	9 (0.5%)	5 (0.3%)	8 (0.4%)

Stroke or mini stroke/TIA	9 (0.4%)	3 (0.2%)	8 (0.4%)	6 (0.3%)

Atrial Fibrillation	7 (0.3%)	7 (0.4%)	7 (0.4%)	2 (0.1%)

Heart attack	5 (0.2%)	4 (0.2%)	6 (0.3%)	6 (0.3%)

Deep vein thrombosis (DVT, ‘Clot in leg’)	3 (0.1%)	0	3 (0.2%)	4 (0.2%)

Other	28 (1.4%)	14 (0.7%)	15 (0.8%)	14 (0.7%)


More than half of the participants (55.8%) reported at least one long COVID symptom at 1-month after discharge. The most reported symptoms were fatigue (39.5%), anxiety (17.9%), breathlessness (10.6%), problems with memory, concentration, or decision making (8.5%), chest pain (8.4%), and palpitations (8.3%). Very few participants reported anosmia or no sense of smell (0.7%), and ageusia or no sense of taste (1.2%). At three-month follow up, over a third of the participants (36.7%) reported experiencing at least one long COVID symptoms, with fatigue (22.5%), anxiety (10.8%), and memory problems (14.9%) being the most commonly reported. Further, a quarter of the participants experienced at least one long COVID symptom during the 9–12-month follow-up period. These symptoms included fatigue (14.5%), anxiety (7.8%), memory problems (8.6%), and palpitations (8.5%). 45.2% of participants reported moderate to severe fatigue at 1-month, while 22.8% reported the same at 9–12-month follow-up (Table 2).

Figure B illustrates the number of persistent long COVID symptoms reported over a one-year follow-up period. At the 1-month follow-up, one fifth of the participants (19.4%) experienced three or more long COVID symptoms, which decreased to half, i.e. 11.5% at the one-year follow-up. In terms of the new onset of diseases following discharge, 10.8% of the participants reported the occurrence of at least one new disease at one-month. This subsequently decreased to 4.6%, 3.3%, and 2.2% at the 3-, 6-, and 9–12-month follow-ups, respectively. The most commonly reported new illnesses were pulmonary embolism (7.6%, n = 173/2274), kidney diseases (4.0%, n = 91/2274), and hypertension (3.1%, n = 71/2274) (Table 2).

#### Mortality, MACE, and long COVID symptoms trends during follow-up

**Figure 2:**
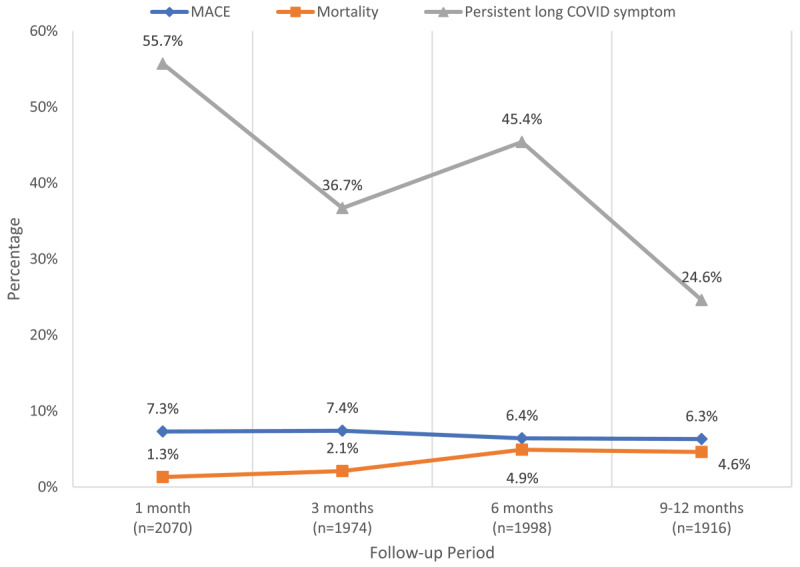
Trends in mortality, MACE, and at least 1 persistent Long COVID symptom over the follow-up period.

Figure 2 shows the trends in mortality, major adverse cardiovascular events (MACE), and persistent long COVID symptoms during a one-year follow-up after discharge. The mortality rate increased over the follow-up period. At 1-month, 1.3% (n = 28) of individuals died, followed by 2.1% (n = 41), 4.9% (n = 98), and 4.6% (n = 87) reported deaths at the 3-, 6-, and 9–12-month follow-ups, respectively. The new onset MACE outcomes declined marginally from 7.3% at 1-month to 6.3% at 9–12-month. The persistent long COVID symptoms, also declined over time, with more than half of the participants (55.8%) reporting at least one long COVID symptom at 1-month, vs. 24.5% reporting long COVID symptoms at 9–12 months.

**Figure 3:**
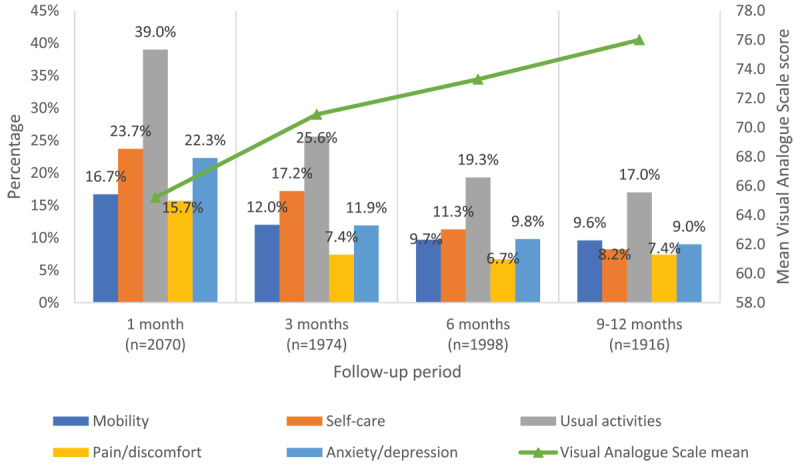
Trends in EQ-5D dimensions over the follow-up period.

On the EQ-5D scale, the most commonly reported difficulty during the follow-up period was in performing usual activities (Figure 3). Regarding participants’ working status, approximately 56% of participants reported changes in their employment status due to COVID-19 infection, most commonly resulting in unemployment, or retirement (supplementary file, Table B).

#### Factors associated with mortality and MACE during follow-up

We found that age was strongly associated with in-hospital mortality (Table 3). For example, individuals older than 70 years had a 14 times higher risk of in-hospital death compared to younger individuals (<50 years) (RR = 13.89 [95% CI: 11.8, 15.98]). This relative risk persisted at the 9–12 month period (RR = 16.61 [95% CI: 12.88, 20.34]). Hispanics had eight times higher risk of death (RR = 8.82 [95% CI: 1.21, 64.32]), followed by 3 times higher risk for blacks (RR = 3.67 [95% CI: 0.48, 28.24]), compared to Caucasian. When comparing WHO regions, patients from Latin America (RR = 2.39 [95% CI: 1.42, 4.03]) had twice increased risk of mortality and Sub-Saharan Africa (RR = 1.21 [95% CI: 0.64, 2.29]) had 21% higher risk of death compared to the European region. Further, participants with pre-existing coronary heart disease, renal disease, COPD, diabetes, and smokers had higher risk of death compared to those without these comorbidities and non-smokers, respectively.

**Table 3:** Factors associated with mortality using Generalized Estimating Equation (GEE) models.


FACTORS	DEATH						RR* [95% CI]

	IN-HOSPITAL (N = 128)	1 MONTH (N = 28)	3 MONTH (N = 41)	6 MONTH (N = 98)	9–12 MONTH (N = 87)	OVERALL (N = 382)	

	MARGINAL ESTIMATES						

**Overall**	4.40(3.74,5.06)	1.52(1.0,2.08)	2.36(1.63,3.09)	5.22(4.19,6.24)	4.74(3.82,5.66)	3.63 (3.28,3.98)	

**Age, years**							

<50	0.62(0.35,0.9)	0.22(0.09,0.35)	0.34(0.16,0.52)	0.83(0.47,1.19)	0.74(0.42,1.07)	0.56(0.33,0.79)	1

51–60	1.14(0.6,1.68)	0.4(0.16,0.65)	0.63(0.29,0.96)	1.52(0.82,2.23)	1.36(0.74,1.98)	1.02(0.57,1.47)	1.83 (1.00,3.33)

61–70	2.92(2.11,3.73)	1.04(0.59,1.48)	1.61(0.98,2.25)	3.92(2.76,5.07)	3.5(2.42,4.57)	2.62(1.97,3.28)	4.69 (2.89,7.61)

>70	13.89(11.8,15.98)	4.94(3.16,6.72)	7.67(5.36,9.98)	18.61(15.1,22.12)	16.61(12.88,20.34)	12.46(11.19,13.72)	22.30 (14.29,34.80)

**Gender**							

Male	4.86(4.05,5.67)	1.69(1.06,2.33)	2.63(1.8,3.46)	5.85(4.59,7.11)	4.55(3.5,5.61)	4.04(3.54,4.55)	1

Female	3.73(2.98,4.48)	1.3(0.79,1.81)	2.02(1.33,2.7)	4.49(3.47,5.51)	3.5(2.63,4.37)	3.1(2.61,3.6)	0.77 (0.63,0.94)

Ethnicity**							

Caucasian	2.13(–2.07,6.32)	0.62(–0.66,1.89)	0.95(–0.94,2.85)	2.19(–2.14,6.51)	1.92(–1.88,5.72)	1.58(–1.54,4.7)	1

Hispanic	18.76(13.33,24.19)	5.44(3.07,7.81)	8.42(5.17,11.66)	19.29(13.61,24.98)	16.93(12.09,21.78)	13.95(10.51,17.4)	8.82(1.21,64.32)

Black	7.8(3.61,11.99)	2.26(0.82,3.7)	3.5(1.35,5.66)	8.02(3.7,12.34)	7.04(3.13,10.96)	5.8(2.77,8.84)	3.67(0.48,28.24)

Middle Eastern	3.93(1.66,6.19)	1.14(0.4,1.88)	1.76(0.69,2.84)	4.04(1.76,6.31)	3.54(1.54,5.55)	2.92(1.32,4.52)	1.85(0.24,14.32)

Asian	4.18(3.54,4.83)	1.21(0.77,1.66)	1.88(1.31,2.45)	4.3(3.39,5.21)	3.78(2.91,4.65)	3.11(2.75,3.47)	1.97(0.27,14.21)

Other	4.25(2.51,5.99)	1.23(0.53,1.94)	1.91(0.91,2.9)	4.37(2.57,6.18)	3.84(2.23,5.45)	3.16(1.91,4.41)	2.00 (0.27,14.95)

Region**							

Europe	5.5(2.87,8.13)	1.57(0.6,2.54)	2.41(1.02,3.79)	5.57(2.9,8.24)	4.92(2.52,7.32)	4.05(2.17,5.93)	1

Asia Pacific	3.81(2.75,4.87)	1.09(0.62,1.55)	1.67(0.98,2.36)	3.86(2.72,4.99)	3.41(2.35,4.46)	2.81(2.1,3.51)	0.69(0.41,1.18)

Latin America	13.16(9.21,17.11)	3.76(2.14,5.38)	5.76(3.58,7.95)	13.33(9.53,17.12)	11.78(8.55,15.01)	9.69(7.34,12.05)	2.39(1.42,4.03)

Middle East	4.01(1.69,6.33)	1.15(0.4,1.89)	1.76(0.68,2.83)	4.07(1.78,6.35)	3.59(1.56,5.62)	2.96(1.34,4.57)	0.73(0.36,1.5)

South East Asia	4.33(3.62,5.05)	1.24(0.78,1.7)	1.9(1.33,2.46)	4.39(3.4,5.37)	3.88(2.94,4.82)	3.19(2.78,3.61)	0.79(0.49,1.28)

Sub Saharan Africa	6.67(3.73,9.61)	1.91(0.78,3.03)	2.92(1.31,4.53)	6.76(3.66,9.85)	5.97(3.12,8.83)	4.92(2.77,7.07)	1.21(0.64,2.29)

Income group**							

LIC	9.24(0,18.48)	2.58(–0.11,5.27)	3.95(–0.17,8.06)	9.2(0,18.4)	8.36(0.04,16.68)	6.76(0.08,13.44)	1

LMIC	4.39(3.67,5.1)	1.22(0.77,1.68)	1.87(1.31,2.43)	4.37(3.41,5.32)	3.97(3.03,4.9)	3.21(2.82,3.6)	0.47(0.18,1.28)

UMIC	7.64(5.66,9.62)	2.13(1.24,3.02)	3.26(2.08,4.45)	7.61(5.79,9.43)	6.91(5.28,8.55)	5.59(4.5,6.69)	0.83(0.3,2.27)

HIC	4.36(2.97,5.74)	1.22(0.67,1.76)	1.86(1.06,2.66)	4.34(2.93,5.74)	3.94(2.6,5.29)	3.19(2.27,4.11)	0.47(0.17,1.32)

**Smoking status**							

Never	3.36(2.72,4.01)	1.17(0.71,1.64)	1.82(1.2,2.44)	4.09(3.18,4.99)	3.16(2.37,3.95)	2.8(2.36,3.23)	1

Current smoker	2.37(1.16,3.58)	0.83(0.3,1.35)	1.28(0.58,1.99)	2.88(1.43,4.34)	2.23(1.08,3.39)	1.97(1.01,2.94)	0.71(0.42,1.18)

Former smoker	6.86(5.41,8.31)	2.39(1.45,3.33)	3.71(2.45,4.97)	8.34(6.2,10.48)	6.45(4.65,8.25)	5.71(4.65,6.77)	2.04(1.59,2.61)

Unknown	5.55(4.11,6.98)	1.93(1.13,2.73)	3(1.88,4.11)	6.74(4.78,8.7)	5.22(3.75,6.69)	4.61(3.58,5.65)	1.65(1.24,2.19)

**Pre-existing conditions**							

**Hypertension**							

Yes	5.56(4.68,6.44)	1.92(1.2,2.64)	2.98(2.04,3.91)	6.54(5.15,7.93)	5.19(3.96,6.42)	4.58 (4.03,5.14)	1.77 (1.43,2.19)

**Coronary Artery Disease**							

Yes	7.45(5.62,9.29)	2.6(1.53,3.66)	4.05(2.58,5.53)	8.97(6.41,11.54)	6.98(4.78,9.18)	6.20 (4.80,7.60)	1.88 (1.45,2.43)

Stroke***							

Yes	8.15(5.02,11.29)	2.19(1.07,3.31)	3.37(1.78,4.96)	7.95(4.86,11.04)	7.36(4.44,10.28)	5.89 (3.78,8.01)	1.67 (1.15,2.42)

Heart failure***							

Yes	9.71(6.6,12.82)	2.66(1.37,3.95)	4.1(2.27,5.93)	9.66(6.38,12.94)	8.93(5.81,12.06)	7.11 (4.96,9.26)	2.07 (1.50,2.85)

Renal disease***							

Yes	9.51(7.25,11.78)	2.56(1.52,3.6)	3.94(2.56,5.33)	9.35(6.94,11.76)	8.71(6.32,11.11)	6.92 (5.54,8.29)	2.17 (1.73,2.73)

**COPD/Asthma**							

Yes	6.97(5.42,8.52)	2.43(1.42,3.44)	3.77(2.45,5.08)	8.27(6.08,10.47)	6.51(4.65,8.36)	5.77 (4.62,6.92)	1.79 (1.41,2.26)

**Diabetes**							

Yes	5.69(4.7,6.68)	1.97(1.21,2.73)	3.05(2.07,4.04)	6.75(5.23,8.27)	5.29(3.97,6.61)	4.70 (4.03,5.38)	1.55 (1.27,1.89)

**Tuberculosis**							

Yes	2.61(–1.12,6.35)	0.9(–0.41,2.22)	1.4(–0.67,3.47)	3.1(–1.35,7.54)	2.42(–1.05,5.89)	2.16 (–0.92,5.23)	0.59 (0.14,2.47)

**HIV**							

Yes	5.72(0.56,10.88)	1.98(0.05,3.91)	3.07(0.21,5.93)	6.8(0.59,13.01)	5.3(0.4,10.21)	4.73 (0.49,8.96)	1.31 (0.53,3.22)

**Oxygen therapy**							

Yes	10.79(8.25,13.34)	3.82(2.29,5.34)	5.97(3.92,8.02)	13.1(9.79,16.41)	10.36(7.48,13.25)	8.92 (7.19,10.65)	3.76 (2.92,4.84)

**ICU admission**							

Yes	12.91(9.89,15.94)	3.78(2.15,5.42)	5.8(3.7,7.91)	13.38(10.02,16.74)	12.35(9.12,15.57)	9.76 (7.82,11.70)	3.23 (2.57,4.06)

BMI categories***							

Normal 18–24	4.78(2.26,7.3)	1.88(0.6,3.16)	3.19(1.31,5.08)	9.55(4.65,14.45)	8.29(3.89,12.7)	5.43(2.76,8.1)	1

Underweight <18	2.78(2.05,3.51)	1.09(0.59,1.59)	1.86(1.17,2.55)	5.55(4.32,6.79)	4.82(3.65,6)	3.16(2.67,3.64)	1.72(1.02,2.89)

Overweight 25–29	2.13(1.48,2.77)	0.83(0.43,1.24)	1.42(0.83,2.01)	4.25(2.96,5.54)	3.69(2.54,4.83)	2.41(1.83,3)	0.76(0.57,1.02)

Obese ≥30	2.22(1.11,3.34)	0.87(0.36,1.38)	1.48(0.64,2.33)	4.44(2.35,6.53)	3.86(1.95,5.76)	2.52(1.39,3.65)	0.8(0.49,1.31)


*RR for mortality over time for each covariate is calculated by keeping the site name as a fixed effect variable. **For variables ethnicity, region, and income group, the analysis did not include site name because of correlation. ***For variables stroke, heart failure, CKD and BMI categories, the models with site as fixed effect did not converge, hence site was excluded from the model.

Factors associated with long COVID symptoms and new onset diseases over the one-year follow-up period are presented in supplementary file, Table C. The most commonly reported long COVID symptoms during any follow-up assessment were fatigue, anxiety, and breathlessness. At one-month, the risk of experiencing fatigue and breathlessness were significantly higher, which declined by half at 9–12 months.

Factors associated with major adverse cardiovascular events (MACE) during hospitalization, follow-ups periods, and overall MACE outcomes are presented in supplementary file, Table D. The elderly participants (>70 years) compared to younger participants (<50 years) had seven times higher risk of MACE over the study follow-up duration. Former smokers had a higher risk (RR = 1.77 [95% CI: 1.50, 2.08]), compared to non-smokers. Like mortality, pre-existing cardiovascular comorbidities (hypertension, coronary artery disease, stroke, and heart failure) and non-cardiovascular comorbidities (renal diseases, COPD, and diabetes) were significantly associated with a higher risk of MACE outcomes (p-value < 0.001). ICU admission and oxygen therapy were positively associated with a doubled risk for MACE outcomes. In contrast to mortality findings, obese participants (BMI ≥30) were twice as likely to develop MACE outcomes.

## Discussion

The WHF COVID-19 long-term follow-up study is one of the largest multi-country studies, including LMICs, reporting on the long-term follow-up of COVID-19 hospitalized patients. Most of the participants contracted the omicron variant of the SARS-CoV-2 virus and were followed for up to a one-year period post-discharge. At the time of hospital admission, 55% of participants reported receiving at least two doses of COVID-19 vaccination.

Most of the participants (56%) experienced at least one long COVID symptom after one month, but this decreased to half (25%) during the follow-up at 9–12 months. The most commonly reported long COVID symptoms during any follow-up assessment were fatigue, anxiety, and breathlessness. Although the participants reported improved health and reduced long COVID symptoms over time, about a quarter of patients reported at least one persistent long COVID symptom after one year. A longitudinal cohort study from China showed that participants reported significant problems with mobility, usual activity, pain/discomfort, and anxiety/depression at six-month follow-up (P < 0.001). 31.5% of participants (out of 856 long COVID patients) reported persistent long COVID symptoms at the one-year follow-up (12). Another retrospective study from India reported long COVID symptoms in 8.2% of participants, with fatigue and cough being the most commonly reported symptoms (13). Although these studies indicated a low incidence of long COVID among individuals infected with the Omicron variant, a gradual improvement in their overall health, and a reduction in symptom severity, the proportion of affected patients (10%) remains significant, thus necessitating an urgent need for long COVID treatment and management protocols.

We observed a substantial proportion of mortality after hospital discharge, with a total mortality of 15% within one year even with high COVID vaccination rates. Approximately, one-third of the non-survivor participants were elderly individuals (>70 years), and 62% were male. A multi-center retrospective study conducted in the United States also reported a high mortality rate of 27% for the Omicron COVID strain, and 17% of patients developed in-hospital cardiovascular events. The mean (SD) age was 62.4 (14) years, 63% were male, and patients were hospitalized for COVID-19 pneumonia. Pre-exiting comorbidities reported were hypertension (52%), hyperlipidemia (38%), heart failure (20%), and diabetes (38%) (14). We have also observed that older individuals (>70 years), being underweight (BMI < 18 kg/m^2^), having pre-existing comorbidities, and ICU admission were significantly linked to mortality. Similarly, a pan-India, longitudinal, prospective study conducted in 14 hospitals demonstrated that age >65 years, pre-existing comorbidities, and lack of vaccination were the independent risk factors for mortality, regardless of the COVID virus strain (15). Our findings are also consistent with broader evidence that individuals with pre-existing comorbidities are at greater risk for severe COVID-19 outcomes, as summarized in the CDC’s updated review of underlying conditions (16).

In terms of clinical complications, the new onset diseases observed were pulmonary embolism (7.6%), kidney diseases (4.0%), and hypertension (3.1%). The observed incidence of pulmonary embolism in our cohort aligns with known pathophysiological mechanisms of COVID-19–associated endotheliopathy. SARS-CoV-2 spike protein binding to ACE2 receptors on endothelial cells has been shown to disrupt vascular homeostasis and promote pro-thrombotic states by impairing local clotting regulation and inducing inflammation. These processes likely contribute to the heightened risk of thrombotic events, such as pulmonary embolism, particularly in hospitalized patients (17). Other studies have also reported higher proportion of pulmonary embolism, 8.3% to 23.8% in COVID patients with Omicron strain (18,19). Patients with new or ongoing dyspnea showed a higher proportion (25/105 patients) of pulmonary embolism in Turkey, with a mean (SD) age of 55.4 (66.4) years, 32% male, and mean (SD) BMI of 30.7 (6.2) (20). A retrospective multi-centre COVID-19 observational study in France demonstrated that male gender, a history of stroke, atrial fibrillation, dyspnoea was significantly associated with the occurrence of pulmonary embolism (103/1240 patients) (21). A multi-centre prospective study in the UK highlighted that COVID survivors commonly presented with lung, kidney, and brain abnormalities at the 5-month follow-up (n = 259). These survivors were older, obese, and had more comorbidities (22). A significant number of participants (24.1%) reported major adverse cardiovascular events during one-year follow-up period, and being obese (BMI >30 kg/m^2^) was found to be positively associated. These findings align with other longitudinal cohort studies (13,21,23) and are crucial in identifying high-risk patients who require careful monitoring to prevent further complications following discharge.

Recent large-scale online surveys conducted in China and UK present long-term consequences of COVID-19 infection. For example, a study of 74,075 Chinese participants revealed that 10–30% of participants experienced long COVID symptoms (18) and data of 242,712 participants from England revealed that 7.5% of participants experienced persistent Long COVID symptoms up to three months, while 5.2% of participants experienced symptoms at 12 months (19). Both studies found that the severity of acute illness, pre-existing comorbidities, and female gender were risk factors for long COVID symptoms. Our study findings were comparable to data reported from China (10–30%), however, lower rates of long COVID symptoms observed in England study (5.2%) could be attributed to differences in active surveillance, better equipped healthcare system and population risk profile. Further, it is unclear whether the online survey participants have had any prior hospitalization due to COVID-19 infection, which is an important predictor of disease severity and long-term adverse consequences.

## Strengths and Limitations of Study

The major strength of the study is large cohort with diverse representation across various countries from different regions. Other important strengths were consecutive enrolment of hospitalized, and RT-PCR diagnosed COVID-19 patients, use of standardized tools, long-term follow-up over one year, and high follow-up rate (90%). However, there are a few limitations to consider. First, the study participants consisted of individuals who had been hospitalized due to COVID-19. These groups of patients represent individuals with more severe COVID-19 infections and were considered high-risk. The data may not be applicable to patients with mild COVID-19 infections who received treatment in outpatient facilities. Second, our study cohort included a majority of participants from Southeast Asia and Asia-Pacific regions, with relatively lower representation from other regions. While this reflects the pragmatic site-based recruitment during the pandemic, it may limit the generalizability of findings to underrepresented populations. Additionally, as the sample size was not stratified by region or ethnicity, subgroup analyses should be interpreted with caution. Third, the follow-up data were collected via telephone, which introduces limitations such as self-reported data on COVID symptoms and cause of deaths. Moreover, there was no comparable cohort of patients hospitalized with other respiratory conditions; hence, we could not conclude differences in outcomes due to various respiratory diseases. Fourth, our findings showed comparable mean BMI values between survivors and non-survivors, it is important to note that BMI data were missing for approximately 16% of the study population. This level of missingness introduces uncertainty, particularly as obesity is a well-established risk factor for severe COVID-19 outcomes. While we observed that underweight status was associated with mortality, and obesity with increased MACE risk, the absence of BMI data for a subset of patients may have attenuated these associations or introduced residual confounding. Future studies with more complete anthropometric data are warranted to validate these findings.

## Conclusion

Our data from a global study involving the majority of patients from low- and middle-income countries showed a high burden of persistent long COVID symptoms among hospitalized COVID-19 patients. Our study provides large-scale and long-term observational data concerning the so-called milder variant – Omicron, and from LMICs, as the majority had tested positive for the Omicron COVID-19 strain. After hospital discharge, this population still presents a substantial mortality and morbidity rate, with approximately a quarter of patients reporting at least one persistent long COVID symptom after a year. This also emphasizes that healthcare providers should be empathetic to patients presenting with long-term symptoms such as fatigue, anxiety, and breathlessness. Our study underscores the importance of early identification, treatment, and management of long COVID symptoms and future research should focus on assessing the value of targeted screening for long COVID complications among high-risk COVID-19 patients and plan management strategies for the long COVID condition, particularly in low- and middle-income countries.

## Data Accessibility Statement

All the relevant, de-identified study data will be available on reasonable request to the corresponding author.

## Additional File

The additional file for this article can be found as follows:

10.5334/gh.1452.s1 Supplementary file. Tables A to D and Figures A and B.
