# Supplementary material for: Long COVID Syndrome, Mortality and Morbidity in Patients Hospitalized with COVID-19 From 16 Countries: The World Heart Federation Global COVID-19 Study
Source: Glob Heart. 2025 Aug 1;20(1):66. doi: 10.5334/gh.1452 (PMC12315686; doi:10.5334/gh.1452)

Manuscript Title: Long COVID Syndrome, Mortality And Morbidity In Patients Hospitalized With COVID-19 From 16 Countries: The World Heart Federation Global COVID-19 Study

Supplementary File

Contents

Table A: Comorbidities at baseline by survival status ... 2
Table B: Working status of the participants over the follow-up period ... 3
Table C: GEE analysis estimates for long COVID symptoms and new disease onset over the follow-up period ... 4
Table D: Factors associated with Major Adverse Cardiovascular Events (MACE) using GEE models ... 5
Figure A: COVID-19 Vaccine received at baseline and over the follow-up period ... 8
Figure B: Persistent Long COVID symptoms over the follow-up period ... 8

Table A: Comorbidities at baseline by survival status

	Baseline	Non-survivors	Survivors	p-value
	N=2535	N=382 (15%)	N=2153 (85%)	
**CO-MORBIDITIES: Cardiovascular Diseases**				
Hypertension	1380 (54.4%)	252 (66.0%)	1128 (52.4%)	<0.001
Coronary artery disease	356 (14.0%)	74 (19.4%)	282 (13.1%)	<0.001
Heart Failure	181 (7.1%)	41 (10.7%)	140 (6.5%)	0.002
Stroke	134 (5.3%)	29 (7.6%)	105 (4.9%)	0.078
Atrial Fibrillation	109 (4.3%)	25 (6.5%)	84 (3.9%)	0.018
Peripheral vascular disease	63 (2.5%)	12 (3.1%)	51 (2.4%)	0.49
Valvular disease	63 (2.5%)	7 (1.8%)	56 (2.6%)	0.67
Rheumatic Heart Disease	27 (1.0%)	5 (1.3%)	22 (1.0%)	0.69
Cardiomyopathies	24 (0.9%)	1 (0.3%)	23 (1.1%)	0.012
Congenital heart disease	14 (0.5%)	1 (0.3%)	13 (0.6%)	0.095
Chagas disease	2 (<1%)	0 (0.0%)	2 (1%)	0.84
**CO-MORBIDITIES: Non-Cardiovascular Diseases**				
Diabetes	960 (37.9%)	176 (46.1%)	784 (36.4%)	0.001
Chronic Kidney Disease	328 (12.9%)	88 (23.0%)	240 (11.1%)	<0.001
Chronic Pulmonary Disease	250 (9.9%)	70 (18.3%)	180 (8.4%)	<0.001
Asthma	222 (8.8%)	36 (9.4%)	186 (8.6%)	0.62
Dementia	104 (4.1%)	22 (5.8%)	82 (3.8%)	0.057
Malignant neoplasm (any type, greater than 6 months remission)	102 (4.0%)	16 (4.2%)	86 (4.0%)	0.84
Cerebrovascular disease	87 (3.4%)	18 (4.7%)	69 (3.2%)	0.091
Malignant neoplasm (any type, active in the past 6 months)	69 (2.7%)	26 (6.8%)	43 (2.0%)	<0.001
Chronic neurological disorder	58 (2.3%)	13 (3.4%)	45 (2.1%)	0.29
Mental health disorder	57 (2.2%)	11 (2.9%)	46 (2.1%)	0.40
Autoimmune disease	49 (1.9%)	10 (2.6%)	39 (1.8%)	0.081
Cancer on chemotherapy	44 (1.7%)	14 (3.7%)	30 (1.4%)	0.005
Chronic immunosuppression	42 (1.7%)	11 (2.9%)	31 (1.4%)	0.037
Tuberculosis (previous)	35 (1.4%)	4 (1.0%)	31 (1.4%)	0.58
Chronic liver disease	30 (1.2%)	11 (2.9%)	19 (0.9%)	0.003
HIV	25 (1.0%)	5 (1.3%)	20 (0.9%)	0.20
Renal replacement therapy	22 (0.9%)	8 (2.1%)	14 (0.7%)	0.013
Tuberculosis (active)	20 (0.8%)	2 (0.5%)	18 (0.8%)	0.68
Immunodeficiency	12 (0.5%)	3 (0.8%)	9 (0.4%)	0.53
Previous organ transplant	10 (0.4%)	2 (0.5%)	8 (0.4%)	0.35
Hematopoietic stem-cell transplantation (HSCT)	3 (0.1%)	2 (0.5%)	1 (<1%)	0.037
Other	148 (5.8%)	36 (9.4%)	112 (5.2%)	0.001

Table B: Working status of the participants over the follow-up period

	**1 month follow-up**	**3 month follow-up**	**6 month follow-up**	**9-12 month follow-up**
**Working status today**	N=2070	N=1974	N=1998	N=1916
Same as before COVID-19 illness	1686 (81.4%)	1709 (86.6%)	1595 (79.9%)	1552 (81.0%)
Different from before COVID-19 illness	116 (5.6%)	67 (3.4%)	123 (6.2%)	107 (5.6%)
Prefer not to say	15 (0.7%)	4 (0.2%)		9 (0.5%)
**Change in working status after COVID-19 illness**				
Unemployed/student/not working due to COVID-19 restrictions	77 (66.4%)	15 (22.4%)	17 (13.8%)	16 (15.0%)
Retired	22 (19.0%)	3 (4.5%)	5 (4.1%)	5 (4.7%)
Full time carer	8 (6.9%)	3 (4.5%)	2 (1.6%)	
Working full-time/part-time	9 (0.8%)	1 (1.5%)	2 (1.6%)	2 (1.9%)
Sick leave/not working due to illness	5 (4.3%)	1 (1.5%)	1 (0.8%)	1 (0.9%)
**Reasons for change in working status**				
Poor health	99 (85.3%)	18 (26.9%)	21 (17.1%)	18 (16.8%)
Sick leave	22 (19.0%)	3 (4.5%)	4 (3.3%)	4 (3.7%)
New caring responsibility	3 (2.6%)			
Other	3 (2.6%)			
Prefer not to say	2 (1.7%)			
Working hours reduced by employer	1 (0.9%)		1 (0.8%)	1 (0.9%)

Table C: GEE analysis estimates for long COVID symptoms and new disease onset over the follow-up period

	**1 month follow-up**	**3 month follow-up**	**6 month follow-up**	**9-12 month follow-up**
**Persistent long COVID symptoms**				
At least one long COVID symptom	56.1(53.9, 58.2)	35.6(33.5, 37.7)	44(41.8, 46.1)	22.7(20.9, 24.6)
Fatigue (worn out/lacking energy or zest)	39.7(37.6, 41.8)	22(20.2, 23.8)	20.4(18.7, 22.2)	13.8(12.3, 15.4)
Feeling more anxious/worrying	17.8(16.2, 19.5)	10.4(9.1, 11.8)	8.6(7.3, 9.8)	7.5(6.3, 8.7)
Breathlessness	10.9(9.6, 12.3)	5(4, 5.9)	3.9(3.1, 4.7)	4.9(3.9, 5.8)
Problems with memory, concentration or decision making	8.4(7.2, 9.6)	14.6(13, 16.1)	31(29, 33)	8.4(7.1, 9.6)
Chest pain	8.3(7.1, 9.5)	4.2(3.3, 5.1)	4.2(3.3, 5.1)	4.9(3.9, 5.9)
Palpitations	8.2(7, 9.4)	4.6(3.7, 5.5)	8.5(7.3, 9.8)	8.4(7.2, 9.6)
Myalgia (muscles aches)	3.9(3.1, 4.7)	2(1.4, 2.5)	2.2(1.6, 2.8)	2.4(1.7, 3)
No sense of taste	1.2(0.7, 1.6)	0.4(0.1, 0.6)	1(0.6, 1.5)	1(0.6, 1.5)
Anosmia (no sense of smell)	0.8(0.4, 1.1)	0.4(0.1, 0.6)	0.2(0, 0.4)	0.2(0, 0.4)
**New onset of disease since discharge**				
Onset of any 1 disease	10.9(9.5, 12.2)	10.1(8.7, 11.4)	9.8(8.5, 11.1)	10.3(8.9, 11.6)
Pulmonary embolism (PE, “Clot in lung”)	5.8(4.8, 6.8)	5.5(4.5, 6.5)	4.4(3.5, 5.3)	4.1(3.2, 4.9)
Kidney problems	2.1(1.5, 2.8)	1.8(1.2, 2.4)	2(1.4, 2.6)	2(1.4, 2.7)
New onset hypertension	1(0.6, 1.5)	0.8(0.4, 1.2)	1.5(1, 2)	1.9(1.3, 2.5)
New onset diabetes	0.8(0.4, 1.2)	0.3(0.1, 0.6)	0.4(0.1, 0.7)	0.5(0.2, 0.9)
Heart Failure	0.6(0.3, 0.9)	0.7(0.3, 1)	0.6(0.3, 0.9)	0.8(0.4, 1.2)
Stroke or mini stroke/TIA	0.5(0.2, 0.8)	0.4(0.1, 0.7)	0.7(0.4, 1)	0.9(0.5, 1.3)
Heart attack	0.3(0.1, 0.5)	0.2(0, 0.4)	0.4(0.1, 0.6)	0.5(0.2, 0.9)
Atrial Fibrillation	0.3(0.1, 0.6)	0.5(0.2, 0.8)	0.4(0.2, 0.7)	0.3(0.1, 0.6)
Deep vein thrombosis (DVT, “Clot in leg”)	0.1(0, 0.3)	-	0.1(0, 0.3)	0.2(0, 0.4)
Other	1.6(1, 2.1)	1.3(0.8, 1.8)	1.2(0.8, 1.7)	1.3(0.8, 1.8)

Table D: Factors associated with Major Adverse Cardiovascular Events (MACE) using GEE models

Factors	MACE						RR* [95% CI]
	In-hospital (N=478)	1 month (N=91)	3 month (N=65)	6 month (N=104)	9-12 month (N=76)	Overall (N=814)	
**Overall**	18.86(17.33,20.38)	6.68(5.65,7.71)	5.57(4.62,6.51)	8.08(6.93,9.23)	6.89(5.84,7.93)	10.11 (9.39, 10.82)	
**Age, years**							
<50	5.49(4.33,6.64)	2.38(1.82,2.95)	1.88(1.41,2.35)	2.79(2.2,3.39)	2.32(1.79,2.86)	3.3(2.67,3.92)	1
51-60	12.04(9.74,14.33)	5.23(3.97,6.49)	4.12(3.08,5.16)	6.13(4.8,7.46)	5.09(3.94,6.23)	7.23(5.94,8.52)	2.19(1.69,2.85)
61-70	17.51(14.94,20.07)	7.6(6.04,9.17)	5.99(4.6,7.39)	8.91(7.2,10.63)	7.4(5.83,8.97)	10.51(9.06,11.97)	3.19(2.51,4.06)
>70	36.99(33.41,40.56)	16.07(12.99,19.15)	12.66(10.02,15.31)	18.84(15.44,22.23)	15.64(12.51,18.76)	22.21(20,24.43)	6.74(5.37,8.45)
**Gender*** **							
Male	21.46(19.43,23.5)	7.56(6.32,8.81)	6.31(5.16,7.46)	9.18(7.74,10.62)	7.8(6.52,9.09)	11.48 (10.45, 12.51)	1
Female	15.55(13.6,17.5)	5.48(4.49,6.47)	4.57(3.71,5.44)	6.65(5.56,7.74)	5.65(4.65,6.65)	8.32 (7.36, 9.28)	0.72 (0.63, 0.84)
**Ethnicity*** *							
Caucasian	38.62(17.37,59.87)	14.48(6.25,22.7)	12.17(5.24,19.1)	17.47(7.62,27.31)	14.86(6.43,23.28)	21.29(9.58,32.99)	1
Hispanic	22.19(16.36,28.02)	8.32(5.84,10.79)	6.99(4.86,9.12)	10.03(7.17,12.9)	8.54(6.06,11.02)	12.23(9.04,15.42)	0.57(0.31,1.06)
Black	24.08(15.02,33.13)	9.02(5.43,12.61)	7.59(4.53,10.64)	10.89(6.63,15.14)	9.26(5.6,12.92)	13.27(8.32,18.22)	0.62(0.32,1.21)
Middle Eastern	6.4(2.47,10.32)	2.4(0.89,3.9)	2.02(0.73,3.3)	2.89(1.08,4.71)	2.46(0.93,4)	3.53(1.36,5.69)	0.17(0.07,0.38)
Asian	16.99(15.41,18.56)	6.37(5.36,7.38)	5.35(4.44,6.27)	7.68(6.54,8.83)	6.54(5.5,7.57)	9.36(8.58,10.15)	0.44(0.25,0.77)
Other	35.94(29.9,41.97)	13.47(10.48,16.46)	11.32(8.68,13.97)	16.25(12.88,19.62)	13.82(10.85,16.79)	19.81(16.48,23.13)	0.93(0.52,1.65)
**Region*** *							
Europe	28.25(20.41,36.09)	10.65(7.38,13.93)	8.86(5.98,11.75)	12.74(8.94,16.53)	10.71(7.48,13.94)	15.54(11.27,19.82)	1
Asia Pacific	13.63(11.09,16.17)	5.14(4.05,6.22)	4.28(3.28,5.27)	6.15(4.88,7.41)	5.17(4.05,6.28)	7.5(6.18,8.81)	0.48(0.35,0.67)
Latin America	31.63(25.78,37.48)	11.93(9.11,14.74)	9.92(7.51,12.34)	14.26(11.11,17.41)	11.99(9.27,14.72)	17.4(14.19,20.61)	1.12(0.8,1.56)
Middle East	6.46(2.5,10.43)	2.44(0.91,3.97)	2.03(0.74,3.32)	2.91(1.08,4.74)	2.45(0.92,3.98)	3.56(1.37,5.74)	0.23(0.12,0.45)
South East Asia	18.09(16.28,19.91)	6.82(5.67,7.98)	5.68(4.67,6.68)	8.16(6.86,9.45)	6.86(5.71,8.01)	9.95(9,10.91)	0.64(0.48,0.86)

Sub Saharan Africa	29.36(22.47,36.24)	11.07(8.01,14.13)	9.21(6.61,11.81)	13.24(9.74,16.73)	11.13(8.12,14.14)	16.15(12.37,19.93)	1.04(0.72,1.49)
**Income group****							
LIC	7.53(-2.67,17.72)	2.83(-1.02,6.67)	2.29(-0.83,5.4)	3.28(-1.17,7.73)	2.72(-0.97,6.42)	4.08(-1.44,9.61)	1
LMIC	18.99(17.21,20.77)	7.13(5.96,8.31)	5.78(4.76,6.79)	8.27(7,9.55)	6.87(5.74,7.99)	10.3(9.4,11.2)	2.52(0.65,9.79)
UMIC	30.19(26.4,33.99)	11.34(9.18,13.5)	9.18(7.31,11.06)	13.15(10.89,15.41)	10.92(8.94,12.9)	16.38(14.37,18.38)	4.01(1.03,15.62)
HIC	3.99(2.42,5.56)	1.5(0.89,2.1)	1.21(0.7,1.72)	1.74(1.02,2.45)	1.44(0.85,2.04)	2.16(1.32,3.01)	0.53(0.13,2.17)
**Smoking status**							
Never	13.64(12.06,15.21)	5.81(4.75,6.86)	4.64(3.75,5.52)	6.84(5.74,7.93)	5.55(4.58,6.52)	8.22(7.37,9.07)	1
Current smoker	11.14(8.02,14.25)	4.74(3.25,6.24)	3.79(2.58,5)	5.59(3.92,7.25)	4.53(3.15,5.91)	6.71(4.87,8.56)	0.82(0.61,1.10)
Former smoker	24.1(21.13,27.06)	10.26(8.26,12.26)	8.2(6.49,9.9)	12.09(9.83,14.35)	9.8(7.86,11.75)	14.52(12.72,16.33)	1.77(1.50,2.08)
Unknown	16.57(13.15,19.99)	7.05(5.37,8.74)	5.64(4.18,7.1)	8.31(6.28,10.34)	6.74(5.14,8.34)	9.99(8.01,11.96)	1.22(0.97,1.53)
**Pre-existing conditions**							
**Hypertension**							
Yes	21.63(19.66,23.6)	9.2(7.58,10.81)	7.31(5.91,8.7)	10.81(9.06,12.56)	8.72(7.2,10.25)	12.96 (11.87, 14.05)	2.19 (1.87, 2.56)
**Coronary Artery Disease**							
Yes	27.94(23.58,32.31)	12.22(9.58,14.86)	9.8(7.61,11.99)	14.42(11.41,17.42)	11.65(9.14,14.15)	16.88 (14.30, 19.46)	1.92 (1.62, 2.29)
**Stroke**							
Yes	30.53(23.48,37.58)	13.03(9.32,16.73)	10.4(7.37,13.44)	15.36(11.18,19.54)	12.42(8.97,15.88)	18.36 (14.10, 22.63)	1.94 (1.52, 2.46)
**Heart failure**							
Yes	34.95(27.71,42.18)	15.14(11.22,19.05)	12.04(8.79,15.29)	17.8(13.31,22.3)	14.31(10.6,18.01)	20.96 (16.65, 25.26)	2.31 (1.86, 2.87)
**Renal disease*****							

Yes	31.13(26.4,35.86)	11.13(8.81,13.45)	9.29(7.23,11.34)	13.49(10.83,16.16)	11.56(9.19,13.93)	16.78 (14.24, 19.32)	1.83 (1.54, 2.17)
**COPD/Asthma**							
Yes	23.85(20.36,27.34)	10.29(8.15,12.42)	8.25(6.46,10.03)	12.1(9.74,14.45)	9.84(7.84,11.84)	14.47 (12.40, 16.56)	1.61 (1.37, 1.90)
**Diabetes**							
Yes	21.54(19.32,23.77)	9.23(7.57,10.88)	7.38(5.96,8.81)	10.88(9.06,12.7)	8.76(7.21,10.31)	13.01 (11.73, 14.29)	1.66 (1.45, 1.91)
**Tuberculosis**							
Yes	7.05(-0.2,14.3)	3.01(-0.11,6.13)	2.41(-0.1,4.92)	3.53(-0.13,7.19)	2.87(-0.11,5.84)	4.25 (-0.12, 0.09)	0.43 (0.15, 1.20)
**HIV*** **							
Yes	19.46(6.54,32.37)	6.77(2.16,11.39)	5.62(1.79,9.46)	8.24(2.68,13.8)	6.99(2.22,11.76)	10.34 (3.47, 17.23)	1.03 (0.53, 2.01)
**Oxygen therapy**							
Yes	27.95(23.32,32.58)	12.16(9.44,14.89)	9.71(7.41,12.01)	14.39(11.31,17.47)	11.57(8.97,14.18)	16.74 (14.04, 19.44)	2.02 (1.66, 2.45)
**ICU admission*****							
Yes	38.99(33.64,44.34)	14.69(11.67,17.7)	12.26(9.59,14.93)	17.51(14.22,20.8)	14.95(11.99,17.91)	21.46 (18.46, 24.47)	2.41 (2.06, 2.83)
**BMI categories*****							
Underweight <18	10.17(4.56,15.77)	3.93(1.71,6.15)	3.18(1.35,5.01)	4.95(2.16,7.74)	4(1.74,6.26)	5.6(2.53,8.67)	1
Normal 18-24	15.8(13.95,17.65)	6.11(5,7.22)	4.94(3.98,5.9)	7.69(6.41,8.97)	6.22(5.08,7.36)	8.7(7.77,9.63)	1.55(0.89,2.72)
Overweight 25-29	16.5(13.85,19.16)	6.38(5.04,7.72)	5.16(4,6.32)	8.03(6.4,9.67)	6.5(5.13,7.86)	9.09(7.69,10.48)	1.62(0.92,2.87)
Obese ≥30	24.62(19.02,30.21)	9.52(7.01,12.03)	7.7(5.51,9.88)	11.99(9.03,14.94)	9.69(7.17,12.22)	13.56(10.57,16.54)	2.42(1.34,4.37)

Note: MACE includes heart attack, heart failure, atrial fibrillation, stroke, deep vein thrombosis, pulmonary embolism, and CVDs related deaths
**RR for MACE over time for each covariate is calculated by fixing the site name variable*
***For variables ethnicity, region, and income group, the analysis didn't include site name because of correlation*
****For variables sex, renal disease, HIV, ICU admission, and BMI, the models with site name didn't converge, hence site name was excluded*

Figure A: COVID-19 Vaccine received at baseline and over the follow-up period

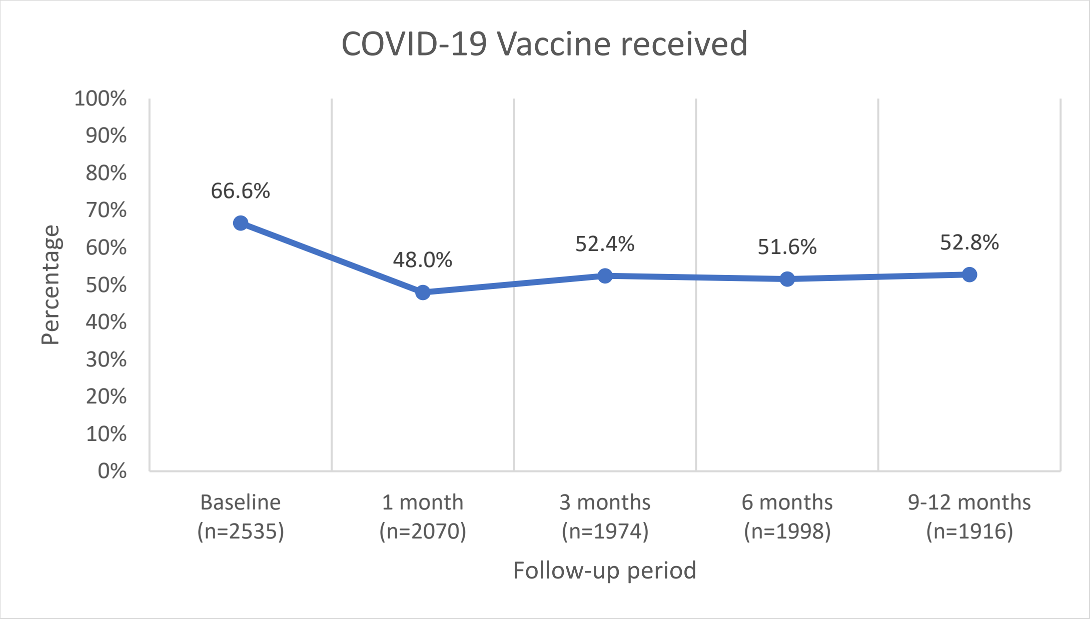

Figure B: Persistent Long COVID symptoms over the follow-up period

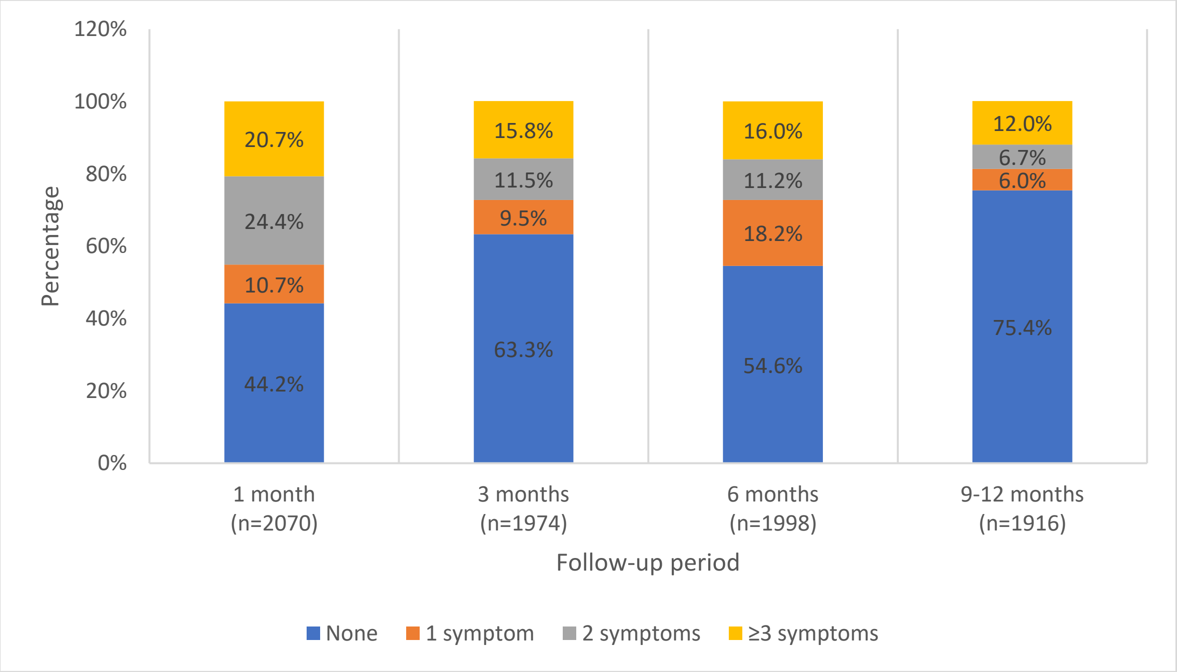

Supplement: Supplementary file. — Tables A to D and Figures A and B. [file gh-20-1-1452-s1.pdf]
